# Effects of Protein Intake on Cognitive Function in Chinese Older Adults: A 10-Year Follow-Up Study

**DOI:** 10.3390/nu17020272

**Published:** 2025-01-13

**Authors:** Ting Zheng, Xiao Zheng, Shujuan Xiao, Benli Xue, Chengyu Chen, Yuyang Li, Xiyan Li, Chichen Zhang

**Affiliations:** 1 School of Health Management, Southern Medical University, Guangzhou 510515, China; 2 Key Laboratory of Philosophy and Social Sciences of Colleges and Universities in Guangdong Province for Collaborative Innovation of Health Management Policy and Precision Health Service, Guangzhou 510515, China; 3 School of Public Health, Southern Medical University, Guangzhou 510515, China; 4 School of Nursing, Southern Medical University, Guangzhou 510515, China

**Keywords:** protein intake frequency, cognitive function, older adults, longitudinal studies, health management

## Abstract

Background: As the global population ages, there is an increasing prevalence of mild cognitive impairment and dementia. Protecting and preserving cognitive function in older adults has become a critical public health concern. Methods: This study utilized data from four phases of the Chinese Longitudinal Healthy Longevity Survey conducted from 2008 to 2018, encompassing a total of 2454 participants. Latent growth curve modeling was employed to analyze the trajectory and role of protein intake frequency and cognitive function. Results: The frequency of protein intake among older adults tends to rise, with individuals exhibiting higher initial levels experiencing smaller subsequent increases. Conversely, cognitive function generally declines, with those starting at higher levels experiencing more pronounced decreases. Notably, the initial frequency of protein intake is positively correlated with the initial level of cognitive function (*β* = 0.227, 95% CI: 0.156 to 0.299, *p* < 0.001), but does not significantly influence the rate of change in cognitive function (*β* = −0.030, 95% CI: −0.068 to 0.009, *p* = 0.128). The rate of change in protein intake frequency is positively associated with the rate of change in cognitive function (*β* = 0.152, 95% CI: 0.023 to 0.280, *p* = 0.020). Conclusions: The alterations in protein intake frequency are linked to alterations in cognitive function among older adults. Maintaining a stable high frequency of protein intake or increasing the frequency of protein intake may contribute to stabilizing cognitive function as well as reducing the risk of cognitive impairment and dementia in older adults.

## 1. Introduction

As the global population ages, the number of older individuals continues to rise, leading to an upward trend in age-related cognitive impairment [1]. In China, the prevalence of dementia has reached figures comparable to those observed in high-income nations and is anticipated to escalate further [2]. Evidence suggests that the prevalence of mild cognitive impairment (MCI) in Chinese older adults is about 12.2% [3], and the prevalence of dementia is about 5.6% [4]. The advancement of dementia significantly heightens risk of various adverse outcomes, including illness, depression, disability, hospitalization, and increased mortality [5]. This rising incidence presents a considerable burden on individuals, families, and health and social care systems [6].

It is estimated that between 10% and 20% of individuals diagnosed with MCI progress to dementia annually, with as many as 50% making this transition within five years [7,8]. MCI is recognized as a transitional stage between normal aging and dementia, and targeting interventions at this population is increasingly recognized as an effective strategy for dementia prevention [9]. The World Health Organization has emphasized the importance of prioritizing the preservation of normal cognitive function in older adults [10]. China has officially become an aging society, and it is projected that by 2050, individuals aged 65 and older will constitute 26.1% of the total population [11]. Consequently, focusing on and sustaining cognitive health within this demographic has emerged as a critical public health objective, essential for preventing the onset of dementia and enhancing the overall well-being of older individuals.

The etiology of dementia is complex and multifactorial; however, it is estimated that up to one-third of dementia cases may be preventable [12]. Approximately 40% of dementia cases may be linked to modifiable risk factors, such as dietary patterns and nutritional status [13]. Some studies over the past few decades have identified various risk factors for dementia and mild cognitive impairment, including poverty, unhealthy lifestyles, and medical conditions [14]. There is ongoing interest in the impact of protein intake on cognitive function, although the evidence is not yet conclusive. Some studies have identified a positive correlation between protein consumption and cognitive function [15,16], suggesting that adequate protein intake may enhance memory and reduce the risk of cognitive impairment [17]. Essential amino acids are critical for normal body function and growth and must be obtained through the diet. The optimal amino acid composition of dietary protein intake may help optimize amino acid metabolism and reduce the risk of dementia [18]. Conversely, other research has failed to establish a significant relationship between protein intake and cognitive performance [19,20,21]. Thus, further exploration of the association between protein intake and cognitive function is warranted.

Protein is an essential nutrient crucial for maintaining normal bodily functions [22,23]. As individuals age, their rates of protein synthesis and catabolism decline, potentially increasing their protein requirements [24]. However, older adults frequently experience diminished appetite and energy intake, which can result in malnutrition [25]. Many older individuals still face inadequate protein intake [26]. Older adults with inadequate protein intake show increased β-amyloid deposition in the brain [27]. This β-amyloid accumulation can impair the functioning of critical brain regions, including the parietal, frontal, and temporal lobes, leading to cognitive decline [28]. Thus, adequate dietary protein intake may play a protective role in maintaining cognitive function. Furthermore, most cohort studies investigating the relationship between protein intake and cognitive function have relied solely on baseline protein intake measurements to assess the risk of cognitive impairment at follow-up [15], neglecting the dynamic nature of protein intake over time, which may introduce measurement errors. Therefore, it is imperative to examine the trajectory of protein intake from a dynamic perspective and to investigate how these changes influence cognitive function, thereby improving the management of dementia risk.

For the reasons mentioned above, this study concentrated on the dynamic alterations in protein intake and utilized data from the Chinese Longitudinal Healthy Longevity Survey (CLHLS) to analyze the evolution of protein intake and cognitive function over a ten-year period among older adults. The CLHLS enabled the examination of the trajectories of changes in both protein intake and cognitive function, as well as the impact of protein intake changes on cognitive performance. Given the rapid aging of the population in China, this research aims to enhance our understanding of the relationship between protein intake patterns and cognitive function in older adults. This understanding is crucial for promoting increased protein consumption among older individuals, thereby improving their cognitive health, reducing the risk of MCI and dementia, and alleviating the associated social burden.

## 2. Materials and Methods

### 2.1. Study Design and Sample

The Chinese Longitudinal Healthy Longevity Survey (CLHLS), initiated in 1998 and conducted every 3 to 4 years, is the first longitudinal study focused on the health and longevity of older Chinese adults. This comprehensive survey utilized a multi-stage stratified sampling method to collect data from individuals aged 65 and older across 23 provinces, municipalities, and autonomous regions in China. The survey covered various topics, including basic demographic information, health status, lifestyle factors, socioeconomic background, and so on [29]. Ethical approval for the survey was granted by the Biomedical Ethics Committee of Peking University, China (IRB00001052-13074), and informed consent was obtained from all participants or their legal representatives prior to their inclusion in the study. The current research utilized data from four waves of the CLHLS, specifically during the periods of 2008–2009 (T1), 2011–2012 (T2), 2014 (T3), and 2017–2018 (T4), involving a total of 2454 older adults who participated fully across all four survey periods.

### 2.2. Measures

#### 2.2.1. Cognitive Function

Cognitive function among older adults was assessed using the Chinese version of the mini–mental state examination (MMSE), a widely recognized tool for evaluating cognitive function that comprises 24 indicators assessing orientation, reaction, attention and calculation, memory, language comprehension, and coordination in older adults. The MMSE is scored from 0 to 30, with higher scores representing better cognitive functioning, while a score below 24 is generally considered as cognitive impairment [30,31]. The Chinese version of the MMSE has been shown to be useful and reliable [32,33]. The Cronbach’s alpha coefficient for the MMSE in this study was 0.923.

#### 2.2.2. Protein Intake Frequency

To assess the consumption of high-protein foods among older adults, a food frequency questionnaire (FFQ) was employed. Participants were asked, “How often do you currently eat this food?” to ascertain the frequency of their dietary intake. This dietary assessment method has been validated in previous research [34,35]. The high-protein food categories included six types: meat, fish and other aquatic products, soy and its derivatives, nuts, eggs, and dairy products [36]. Each food item was assigned a score based on consumption frequency: a score of 5 for “almost every day”, 4 for “at least once a week”, 3 for “at least once a month”, and 2 for “sometimes”, while “rarely or never” was assigned a score of 1. The total scores were summed to form a protein intake index, ranging from 0 to 30, with higher scores indicating more frequent consumption of high-protein foods among older adults.

#### 2.2.3. Covariates

Covariate variables included age, gender (male and female), educational background (years of schooling), self-assessed health status (good, fair, or poor), self-assessed economic status (good, fair, or poor), marital status (married and living together, married but separated, divorced, widowed, and never married), number of chronic diseases (0, 1, 2, and ≥3), healthy dietary intake (including fresh vegetables, fresh fruits, garlic, tea, vegetable grease, mushroom, or algae), and unhealthy dietary intake (including salt-preserved vegetables, sugar, animal fats). Healthy dietary intake and unhealthy dietary intake were quantified by assigning values to each food item based on the response categories: “almost every day” was assigned a value of 5, “at least once a week” received a value of 4, “at least once a month” was given a value of 3, “sometimes” was assigned a value of 2, and “rarely or never” received a value of 1. The scores were summed to create respective indicators, with higher scores indicating a greater frequency of intake. Notably, gender and educational background were considered stable covariates that did not change over time, whereas self-assessed health, self-assessed economic status, marital status, number of chronic diseases, healthy dietary intake, and unhealthy dietary intake were variables that could change throughout the study period.

### 2.3. Statistical Analysis

The basic characterization of continuous variables was conducted using the mean ± standard deviation, while categorical variables were described through frequencies and percentages. Latent growth curve models (LGCMs) were utilized to examine the trajectories and associations between protein intake frequency and cognitive function in this study. LGCMs are commonly utilized to analyze the development of variables over time in longitudinal studies [37]. They can delineate individual change trajectories and uncover underlying growth trends, providing valuable insights into individual variations over time and allowing for the examination of how different variables influence these changes. LGCMs facilitate a more effective and flexible analysis of complex tracking data, accommodating features such as unequally spaced time points, time-varying covariates, and multivariate growth processes [38]. The LGCM reflects the trajectories of protein intake frequency and cognitive function through two metrics: the intercept (I) factor, which reflects initial scores on protein intake frequency and cognitive function at baseline, and the slope (S) factor, which represents the rate of change across the four observation waves.

Firstly, unconditional linear latent growth models were constructed to describe the respective trajectories of protein intake frequency and cognitive function. Subsequently, conditional linear latent growth models were formulated, incorporating time-invariant covariates (gender and years of schooling) and time-varying covariates (age, marital status, self-assessed economic status, self-assessed health status, number of chronic diseases, healthy dietary intake, and unhealthy dietary intake). This approach aimed to account for the influence of these related factors on latent growth and to investigate the heterogeneity among older adults. The conditional linear latent growth model of cognitive function also included protein intake frequency as a time-varying covariate.

Finally, to enhance the understanding of the relationship between protein intake frequency and cognitive function, a parallel latent growth model was constructed. This model formulated a regression equation of intercept and slope factors to link protein intake frequency to cognitive function. It investigated the impact of the initial level of protein intake frequency on both the baseline level and the rate of change in cognitive function among older adults, as well as the effect of changes in protein intake frequency on the rate of change in cognitive function. Fundamental characterization was conducted utilizing SPSS software version 26.0, while latent growth curve modeling was performed using Mplus software version 8.3. Statistical significance was considered to exist when *p* < 0.05 or the 95% confidence interval (95% CI) did not include zero. All correlations were calculated on the basis of 1000 samples using bias-corrected bootstrap standard errors.

## 3. Results

The results include baseline sample characteristics, growth trajectories of protein intake and cognitive function, an analysis of heterogeneity, and the exploration of parallel latent growth relationships between the trajectories of protein intake and cognitive function.

### 3.1. Sample Characteristics

Among the 2454 participants in the study, 1147 were male (46.7%) and 1307 were female (53.3%). At T1, the mean age of the participants was 75.1 years, with an average of 2.9 years of schooling completed. Nearly 60% of the older adults were married and living with their spouses. Approximately 55.4% of the participants rated their health status as good, and 71.2% reported having an average economic status. Table 1 presents the basic characteristics of the participants.

### 3.2. Growth Trajectories

Unconditional linear models were employed to evaluate the initial cognitive function, frequency of protein intake, and their trajectories over time among older adults. The model fit was evaluated using various indices and acceptable thresholds, including χ^2^, degrees of freedom (*df*), CFI (>0.90), TLI (>0.90), RMSEA (<0.08), and SRMR (<0.08) [39]. As presented in Table 2, the unconditional latent growth model for protein intake frequency demonstrated a satisfactory fit. The initial level of protein intake frequency was 17.860 (*p* < 0.001), while the slope was 0.133 (*p* < 0.01), indicating an increasing trend in protein intake frequency across the four measurement periods. The correlation coefficient between the intercept and slope was −0.508 (95% CI: −0.989 to −0.026; *p* = 0.039), suggesting that higher initial levels of protein intake frequency were associated with smaller subsequent increases.

Similarly, Table 2 demonstrates that the unconditional latent growth model for cognitive function also showed a favorable fit. The initial level of cognitive function was measured at 26.673 (*p* < 0.001), while the slope was −0.353 (*p* < 0.001), indicating a declining trend in cognitive function over the four measurement periods. The correlation coefficient between the intercept and slope was 0.531 (95% CI: 0.203 to 0.860; *p* < 0.001), suggesting that higher initial levels of cognitive function were associated with more pronounced subsequent declines.

### 3.3. Heterogeneity Analysis

Conditional linear latent growth models were constructed, incorporating variables such as gender, years of schooling, age, self-rated health status, self-assessed economic status, and marital status, number of chronic diseases, healthy dietary intake, and unhealthy dietary intake into the models of both protein intake frequency and cognitive functioning. Additionally, the frequency of protein intake was incorporated into the model assessing cognitive functioning. The models for protein intake frequency and cognitive function demonstrated a good fit across various indices (the model of protein intake frequency: $\chi^{2}$ = 211.902, *df* = 93, CFI = 0.974, TLI = 0.965, RMSEA = 0.023, and SRMR = 0.024; the model of cognitive function: $\chi^{2}$ = 195.347, *df* = 105, CFI = 0.961, TLI = 0.947, RMSEA = 0.019, and SRMR = 0.018).

The parameter estimates for the conditional linear latent growth model are presented in Table 3 and Table 4. The results indicate significant heterogeneity in both the frequency of protein intake and cognitive function among older adults.

Regarding the frequency of protein intake, analysis of the time-invariant covariates revealed substantial differences in the initial levels of protein intake frequency among older adults with varying educational backgrounds. Specifically, individuals with higher educational attainment exhibited greater initial intake frequency (*β* = 0.338, 95% CI: 0.251 to 0.426; *p* < 0.001). Gender did not appear to significantly influence the frequency of protein intake among older adults (*p* > 0.05). Among the time-varying covariates, age demonstrated a significant positive effect on protein intake frequency (*β* = 0.045~0.089, *p* < 0.05). Self-rated health (*β* = 0.041~0.079, *p* < 0.05) and economic status (*β* = 0.035~0.176, *p* < 0.05) significantly influenced the frequency of protein intake in all four periods. The better the self-rated health and the more affluent the economic status, the higher the protein intake frequency. In addition, the level of frequency of protein intake was higher in older adults who were married and living with their spouses at T1 (*β* = −0.040, 95% CI: −0.078 to −0.003; *p* = 0.032). At T4, older adults with a higher number of chronic conditions also demonstrated a higher level of protein intake frequency (*β* = 0.040, 95% CI: 0.009 to 0.071; *p* = 0.012).

For cognitive functioning, the analysis of time-invariant covariates indicated that initial levels of cognitive function were higher among older adults who were male (*β* = −0.302, 95%CI: −0.546 to −0.059, *p* = 0.015) and had high levels of schooling (*β* = 0.394, 95%CI: 0.138 to 0.650; *p* = 0.003). Among the time-varying covariates, older age (*β* = −0.257~−0.216, *p* < 0.001), worse self-rated health (*β* = 0.078~0.151, *p* < 0.001), and decreased frequency of protein intake (*β* = 0.051~0.117, *p* < 0.05) were associated with poorer cognitive function. At T2 and T3, better economic status (*β* = 0.038~0.098, *p* < 0.05) and a lower number of chronic diseases (*β* = −0.070~−0.041, *p* < 0.05) were associated with better cognitive function. At T4, older adults who reported a higher frequency of consuming healthy foods displayed better cognitive function (*β* = 0.052, 95%CI: 0.006 to 0.097; *p* = 0.025). Conversely, with the exception of T2, those who consumed unhealthy foods more frequently showed a decline in cognitive function (*β* = −0.059~−0.047, *p* < 0.05).

### 3.4. Parallel Process Latent Growth Association

The relationship between changes in protein intake frequency and cognitive function among older adults was analyzed by constructing a parallel process latent growth model, while controlling for individual characteristic variables. The model exhibited a good fit, with the following statistics: $\chi^{2}$ = 354.210, *df* = 74, CFI = 0.931, TLI = 0.907, RMSEA = 0.039, and SRMR = 0.043.

**Figure 1:**
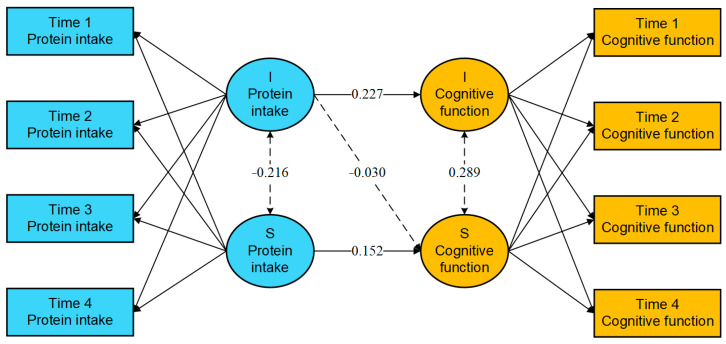
Parameter estimation results of the parallel latent growth curve model. Note: the solid line means statistically significant, and the dashed line means not statistically significant. I—Intercept, S—Slope.

The results shown in Figure 1 indicate that the intercept of protein intake frequency significantly predicts the intercept of cognitive function (*β* = 0.227, 95% CI: 0.156 to 0.299, *p* < 0.001), suggesting that older adults with a higher initial level of protein intake frequency also exhibit higher initial cognitive function. However, the initial level of protein intake frequency did not have a significant effect on the rate of change in cognitive function (*β* = −0.030, 95% CI: −0.068 to 0.009, *p* = 0.128). In contrast, the slope of protein intake frequency was found to significantly and positively predict the slope of cognitive function (*β* = 0.152, 95% CI: 0.023 to 0.280, *p* = 0.020), indicating that a faster rate of increase in protein intake frequency is associated with an accelerated enhancement in cognitive function among older adults.

## 4. Discussion

In this cohort study, a latent growth curve model was employed to evaluate the trajectory of changes in protein intake frequency and cognitive function among older adults, as well as to examine the impact of changes in protein intake frequency on cognitive function changes. The results indicated a general upward trend in protein intake frequency alongside a decline in cognitive function over the ten-year follow-up period. The initial level of protein intake frequency positively influenced the initial level of cognitive function in older adults, but it did not have a statistically significant effect on the rate of change in cognitive function. Conversely, changes in the frequency of protein intake had a significant positive impact on changes in cognitive function.

The current investigation revealed a progressive increase in the frequency of protein intake among older adults, with age exhibiting a significant positive influence on protein intake frequency. This finding is supported by several studies. As individuals age, there is a decline in their ability to synthesize proteins, which results in an increased reliance on externally sourced proteins for energy needs [24,40]. A study across four countries showed that higher protein intake is associated with a reduced incidence of frailty [26]. Dietary protein is essential for preserving skeletal muscle mass and addressing metabolic disorders in older adults, both of which are closely linked to frailty [41]. Frailty may affect the health status of older adults through various mechanisms, such as chronic inflammation and hormonal imbalances, leading to a decline in cognitive function. This association is observed not only in older adults with dementia but also in those without dementia [42]. To mitigate age-related frailty and replenish energy-providing substances, it is likely that older adults will enhance their consumption of protein-dense foods. Moreover, older adults with more years of formal education exhibited higher initial levels in their intake of protein foods. This phenomenon may be attributed to education serving as a significant determinant in the acceptance of sustainable protein sources among older adults [43]. Individuals with higher educational backgrounds typically possess better nutritional knowledge and dietary practices, which makes them more likely to maintain a higher frequency of protein consumption in later life. Interestingly, this study did not identify significant effects of age or gender on the frequency of protein intake among older adults, suggesting that it is equally important to focus on the protein intake among older adults across diverse age groups and genders. Furthermore, self-reported economic status was found to positively influence protein intake frequency during all measurement periods (T1–T4), highlighting the critical role of socioeconomic factors in shaping dietary preferences and food choices among older adults [44]. Therefore, it is essential to ensure that economically disadvantaged older adults receive adequate protein and other vital nutrients.

Consistent with prior research, there is a gradual decline in cognitive functioning among older adults [45]. Our study revealed a negative correlation between age and cognitive function, highlighting age as a critical factor contributing to the rising prevalence of dementia in this population [2]. A meta-analysis indicated that among Chinese older adults aged 60 and above, the prevalence of mild cognitive dysfunction rises by 1.27, 1.45, 1.39, and 1.35 times with every five-year increase in age, respectively [46]. Over four follow-up periods, older adults experienced ongoing aging, accompanied by degeneration of the temporal and frontal lobes and other age-related brain structures [47], leading to declines in cognitive performance. Additionally, we observed a gender effect on the initial levels of cognitive functioning, with females exhibiting lower cognitive function than males at baseline. This discrepancy may be attributed to historically lower educational attainment among females compared to males in earlier periods in China. Self-rated health was positively correlated with cognitive function across all four survey periods. The self-assessed health status of older adults serves as a reflection of their actual health condition, with good health potentially mitigating memory loss and perceptual decline [48], thereby reducing the risk of developing dementia. Consequently, self-assessed health appears to be associated with cognitive function. It is imperative to maintain a long-term focus on the cognitive performance of older adults who are of advanced age and poorer self-assessed health, and to implement relevant cognitive interventions in a timely manner to avert cognitive impairment.

Our findings also indicated that the trajectory of protein intake frequency among older adults was associated with changes in cognitive function. While the initial levels of protein intake frequency did not significantly affect the trajectory of cognitive function changes, the rate of change in protein intake frequency had a positive impact on the rate of change in cognitive function among older adults. Several lines of evidence may help explain this association. Hideaki Sato and colleagues discovered that the addition of essential amino acids can counteract cognitive deficits. They concluded that inadequate protein intake leads to decreased blood amino acid levels, which in turn results in deficiencies in neurotransmitters [49]. Animal-based proteins (including fish, poultry, meat, eggs, and dairy) and plant-based proteins (such as legumes and nuts) are rich in essential amino acids [50]. Sufficient levels of amino acids, particularly methionine, may help reduce the risk of dementia and brain atrophy over time [51]. Additionally, tryptophan is integral to the microbiota of the gut–brain axis, with its metabolites facilitating the development of both the central and enteric nervous systems [52]. The amino acids derived from dietary proteins are metabolized over time [23], and plasma free amino acids reflect nutritional status [53] and may predict changes in cognitive function. Consistent intake of proteins from various sources is more effective in providing sufficient essential amino acids necessary to sustainably influence cognitive function. Therefore, increasing the frequency of protein intake may serve as a preventive measure against cognitive impairment. Future dietary and nutritional interventions aimed at enhancing cognitive function in older adults should promote the sustained intake of high-protein diets. We have also found that the frequency of healthy food consumption has a positive impact on cognitive function in older adults, while more intake of unhealthy foods correlates with a decline in cognitive function. Consequently, to preserve cognitive function in this demographic, it is essential not only to enhance protein intake but also to increase the consumption of other nutritious foods and to minimize the intake of unhealthy options such as pickled vegetables and sugary foods.

The strength of this study lies in its utilization of ten years of national longitudinal data, which ensures that the findings are nationally representative and capture variations in nutritional intake and health status among older adults over an extended period. Additionally, this study examined the trajectories of protein intake frequency and cognitive function in older adults from a dynamic perspective, analyzing the correlation between these two trajectories. Meanwhile, the intake of other healthy foods as well as unhealthy foods was included in the study, which helps to enhance our comprehension of the shifts in nutritional intake and health status among older adults and provides valuable nutritional evidence for the prevention of dementia.

The study also had several limitations that should be noted. Firstly, this study relied on self-reported data from participants, which may have introduced recall bias. Secondly, the sample consisted solely of Chinese older adults who participated in all four phases of the survey simultaneously, potentially leading to sample selection bias. Thirdly, the food frequency questionnaire utilized in the study only assessed the frequency of food intake, preventing the calculation and adjustment for total energy intake. Nevertheless, the reliability and validity of this questionnaire have been established in prior research, and its validity is generally accepted. Lastly, while we endeavored to include both time-varying and time-invariant covariates to control for errors, completely eliminating the influence of confounding factors remains challenging. Future research should employ more precise dietary assessment tools to calculate and adjust for total energy intake and consider a broader range of potential confounding variables to enhance the understanding of nutrition and health among the older adults.

## 5. Conclusions

This study examines the trajectories of protein intake frequency and cognitive function among older adults, as well as the relationships between these two trajectories. The frequency of protein intake among older adults exhibits an upward trend, whereas cognitive function demonstrates a downward trend. Notably, an increased frequency of protein intake may serve as a protective factor for cognitive function in this demographic. The initial level of protein intake affects the initial level of cognitive function in older adults but not its rate of change, whereas the rate of change in the frequency of protein intake affects the rate of change in cognitive function. Therefore, sustaining a consistently high frequency of protein intake or enhancing this frequency may play a significant role in stabilizing cognitive function in older adults.

## Figures and Tables

**Table 1:** Demographic characteristics of participants (*N* = 2454).

Variables	Number (%)	Mean (SD)
Sex		
Male	1147 (46.7)	
Female	1307 (53.3)	
Years of schooling		2.9 (3.7)
Age		75.1 (8.3)
Marital status		
Married	1433 (58.4)	
Separated	71 (2.9)	
Divorced	6 (0.2)	
Widowed	919 (37.5)	
Never married	25 (1.0)	
Self-assessed economic status		
Good	315 (12.8)	
Fair	1747 (71.2)	
Poor	392 (16.0)	
Self-assessed health status		
Good	1359 (55.4)	
Fair	777 (31.7)	
Poor	318 (13.0)	
Protein intake		
T1		17.7 (4.5)
T2		18.1 (4.6)
T3		18.5 (4.5)
T4		17.9 (5.3)
Cognitive function		
T1		26.5 (3.3)
T2		26.4 (3.1)
T3		26.2 (3.3)
T4		25.3 (4.0)

Note: SD—standard deviation.

**Table 2:** Model fit and parameter estimation of unconditional latent growth model.

Variables	$\chi^{2}$ (*df*)	CFI	TLI	RMSEA	SRMR	Mean		r
						**Intercept**	**Slope**	
Protein Intake	66.308 (5)	0.940	0.928	0.071	0.038	17.860 ***	0.133 **	−0.508 *
Cognitive Function	65.903 (5)	0.931	0.917	0.070	0.021	26.673 ***	−0.353 ***	0.531 ***

Note: CFI—comparative fit index, TLI—Tucker–Lewis index, RMSEA—root mean square error of approximation, SRMR—standardized root mean square residual, r—the relevance of intercept and slope; *** *p* < 0.001, ** *p* < 0.01, * *p* < 0.05.

**Table 3:** Parameter estimation results of the conditional latent growth model (time-invariant covariates).

Variables	Protein Intake			Cognitive Function		
	β	95% CI	*p*-Value	β	95% CI	*p*-Value
Intercept						
Sex	0.020	(−0.061, 0.101)	0.627	−0.302	(−0.546, −0.059)	0.015
Years of schooling	0.338	(0.251, 0.426)	<0.001	0.394	(0.138, 0.650)	0.003
Slope						
Sex	−0.002	(−0.176, 0.172)	0.980	−0.106	(−0.444, 0.231)	0.537
Years of schooling	−0.260	(−0.538, 0.018)	0.066	0.242	(−0.349, 0.834)	0.423

**Table 4:** Parameter estimation results of the conditional latent growth model (time-varying covariates).

Variables		Protein Intake				Cognitive Function			
		T1	T2	T3	T4	T1	T2	T3	T4
Age	β	0.045 *	0.047 **	0.089 ***	0.047 **	−0.257 ***	−0.256 ***	−0.254 ***	−0.216 ***
	95% CI	(0.010, 0.080)	(0.020, 0.074)	(0.062, 0.117)	(0.017, 0.077)	(−0.294, −0.220)	(−0.287, −0.225)	(−0.285, −0.222)	(−0.251, −0.182)
Self-assessed health status	β	0.043 *	0.079 ***	0.051 **	0.041 **	0.107 ***	0.078 ***	0.151 ***	0.106 ***
	95% CI	(0.008, 0.079)	(0.044, 0.114)	(0.017, 0.085)	(0.008, 0.074)	(0.068, 0.146)	(0.037, 0.118)	(0.112, 0.191)	(0.064, 0.148)
Self-assessed economic status	β	0.176 ***	0.136 ***	0.061 ***	0.035 *	0.037	0.098 ***	0.038 *	0.019
	95% CI	(0.142, 0.211)	(0.102, 0.169)	(0.028, 0.094)	(0.002, 0.068)	(−0.004, 0.078)	(0.062, 0.134)	(0.001, 0.074)	(−0.018, 0.055)
Marital status	β	−0.040 *	−0.016	−0.018	−0.009	−0.027	−0.004	0.008	−0.051 **
	95% CI	(−0.078, −0.003)	(−0.052, 0.020)	(−0.054, 0.018)	(−0.044, 0.025)	(−0.069, 0.016)	(−0.041, 0.033)	(−0.030, 0.046)	(−0.088, −0.014)
Number of chronic diseases	β	−0.022	0.012	0.018	0.040 *	0.014	−0.041 *	−0.070 **	0.014
	95% CI	(−0.059, 0.014)	(−0.021, 0.046)	(−0.016, 0.052)	(0.009, 0.071)	(−0.021, 0.049)	(−0.077, −0.004)	(−0.110, −0.029)	(−0.050, 0.023)
Healthy diets intake	β	0.346 ***	0.440 ***	0.471 ***	0.493 ***	0.026	−0.013	0.039	0.052 *
	95% CI	(0.310, 0.383)	(0.404, 0.476)	(0.435, 0.507)	(0.456, 0.530)	(−0.017, 0.069)	(−0.058, 0.031)	(−0.002, 0.081)	(0.006, 0.097)
Unhealthy diets intake	β	0.089 ***	0.108 ***	0.129 ***	0.173 ***	−0.059 **	−0.009	−0.052 **	−0.047 *
	95% CI	(0.055, 0.123)	(0.074, 0.142)	(0.096, 0.162)	(0.140, 0.206)	(−0.101, −0.016)	(−0.051, 0.034)	(−0.089, −0.015)	(−0.085, −0.010)
Protein intake	β	-	-	-	-	0.117 ***	0.079 **	0.051 *	0.052 *
	95% CI	-	-	-	-	(0.077, 0.157)	(0.034, 0.125)	(0.006, 0.096)	(0.008, 0.097)

Note: *** *p* < 0.001, ** *p* < 0.01, * *p* < 0.05.

## Data Availability

The raw data supporting the conclusions of this article can be found on the website: https://opendata.pku.edu.cn/dataverse/CHADS (accessed on 20 October 2023).
